# Biliary tract neoplasms: detection and staging CT and Ultrasound

**DOI:** 10.1186/1470-7330-14-S1-O4

**Published:** 2014-10-09

**Authors:** Richard M Gore, Robert I Silvers

**Affiliations:** 1 Department of Radiology, North Shore University Health System, University of Chicago, Pritzker School of Medicine, Evanston, IL USA

## 

Most biliary tract neoplasms are malignant and have been traditionally divided into cancers of the gallbladder, the extrahepatic bile ducts, and ampulla of Vater. Although infrequent, bile duct carcinomas and cancer of the gallbladder are not rare. In the United States, an estimated 6,000 to 7,000 new cases of carcinoma of the gallbladder and 3,000 to 4,000 new cases of carcinoma of the bile ducts are diagnosed annually. Familiarity with the imaging characteristics of gallbladder and bile duct neoplasms is important to expedite diagnosis and appropriate treatment of patients who often present with nonspecific symptoms of right upper quadrant pain, jaundice, and weight loss. This workshop presents the CT and ultrasound features of biliary tract neoplasms important for early diagnosis, staging, and follow-up of these often lethal neoplasms

